# A long term, non-tumorigenic rat hepatocyte cell line and its malignant counterpart, as tools to study hepatocarcinogenesis

**DOI:** 10.18632/oncotarget.14984

**Published:** 2017-02-01

**Authors:** Maria Maddalena Angioni, Kevin Bellofatto, Simone Merlin, Silvia Menegon, Andrea Perra, Annalisa Petrelli, Pia Sulas, Silvia Giordano, Amedeo Columbano, Antonia Follenzi

**Affiliations:** ^1^ Department of Biomedical Sciences, University of Cagliari, 09124 Cagliari, Italy; ^2^ Department of Health Sciences, University of Piemonte Orientale, 28100 Novara, Italy; ^3^ Department of Oncology, University of Torino School of Medicine, Candiolo Cancer Institute-FPO, IRCCS, 10060 Candiolo, Italy

**Keywords:** resistant hepatocyte, CD90.1, CD24, HCC cell lines, immortalized non-tumorigenic cells

## Abstract

Hepatocellular carcinoma (HCC) is the fifth most common cancer worldwide and the second cause of cancer-related death. Search for genes/proteins whose expression can discriminate between normal and neoplastic liver is fundamental for diagnostic, prognostic and therapeutic purposes. Currently, the most used *in vitro* hepatocyte models to study molecular alterations underlying transformation include primary hepatocytes and transformed cell lines. However, each of these models presents limitations. Here we describe the isolation and characterization of two rat hepatocyte cell lines as tools to study liver carcinogenesis. Long-term stable cell lines were obtained from a HCC-bearing rat exposed to the Resistant-Hepatocyte protocol (RH cells) and from a rat subjected to the same model in the absence of carcinogenic treatment, thus not developing HCCs (RNT cells). The presence of several markers identified the hepatocytic origin of both cell lines and confirmed their purity. Although morphologically similar to normal primary hepatocytes, RNT cells were able to survive and grow in monolayer culture for months and were not tumorigenic *in vivo*. On the contrary, RH cells displayed tumor-initiating cell markers, formed numerous colonies in soft agar and spheroids when grown in 3D and were highly tumorigenic and metastatic after injection into syngeneic rats and immunocompromised mice. Moreover, RNT gene expression profile was similar to normal liver, while that of RH resembled HCC. In conclusion, the two cell lines here described represent a useful tool to investigate the molecular changes underlying hepatocyte transformation and to experimentally demonstrate their role in HCC development.

## INTRODUCTION

Primary cultures of normal hepatocytes are widely used to study the biochemical/molecular events involved in cell death, proliferation and differentiation [1, 2]. However, several disadvantages limit their use: 1) high inter-individual variability due to differences of the liver of origin (age, gender, healthy/pathological status, xenobiotic treatment); 2) the ability to retain only for a short period most of their features, such as their proliferative capacity following specific growth factor stimulation or expression of drug/xenobiotic-metabolizing enzymes [3]. Thus, to improve the maintenance of hepatocyte function and the longevity of the cultures, several alternatives have been developed, but still several problems need to be overcome.

Some conditions were identified to improve *in vitro* culture, including continuous medium and oxygen supply, and metabolite removal [4, 5]. In addition, various hollow fiber bioreactor systems were developed using hepatocytes of several species [6]. In these systems, cells attach to the surface of fibers or membranes and reorganize themselves into three-dimensional structures that may result in a hepatocyte microenvironment closely resembling the physiological one. Unfortunately, the described cell culture systems are not yet standardized and cannot be easily transferred to other laboratories.

To overcome limitations that negatively regulate human hepatocyte viability and functionality, isolated rodent hepatocytes have been increasingly used as a tool to identify pharmacological and toxicological responses to drugs. Primary rat hepatocytes represent a useful experimental model as their isolation is a relatively easy procedure, guaranteeing a good success rate and an adequate degree of reproducibility. In addition, this procedure provides a large number of cells from a single rat liver. Nevertheless, their use cannot be exploited for studying the biochemical/molecular events leading to cell transformation, as primary hepatocytes survive in culture no longer than 1 week. Even though long-lasting HCC cell lines are useful for drug screening and/or molecular manipulation of gene expression, a major limit in their use is the lack of a normal counterpart for reference.

In the present study, taking advantage of the Resistant Hepatocyte model of rat hepatocarcinogenesis (R-H) [7], we generated and characterized a long-term, non-tumorigenic hepatocyte cell line (RNT), and the corresponding fully transformed cell line (RH). These matched cell lines represent a valuable model to study hepatocarcinogenesis, through genetic engineering aimed at reproducing the multistep process of liver cancer development.

## RESULTS

### Isolation and characterization of RNT and RH cell lines

The R-H model consists of a single injection of DENA followed by a brief exposure to a promoting environment (2-AAF + PH). HCCs arise 10-14 months after DENA treatment (the protocol scheme is shown in Supplementary Figure 1). Control rats exposed to 2-AAF + PH in the absence of DENA, do not develop tumors. RH and RNT cells were obtained from a rat exposed to the full R-H protocol and from a rat not exposed to DENA, respectively. Briefly, cells were isolated from liver rats through collagenase perfusion by portal vein and maintained in culture. Both cell lines were vital after more than 50 passages in conventional 2D culture dishes, and did not change their morphology and behavior. Therefore, they can be defined as “spontaneously immortalized” cells.

**Figure 1:**
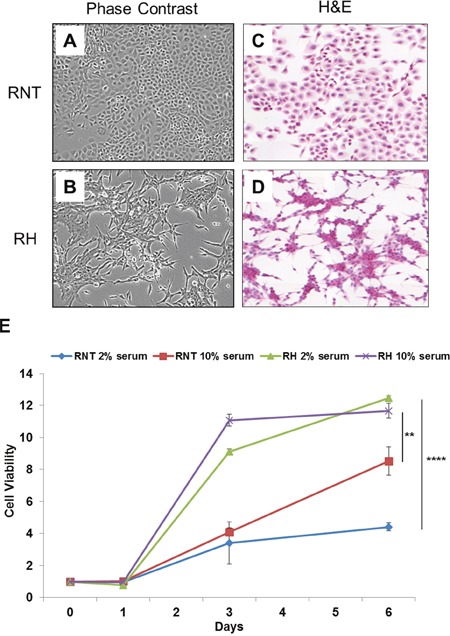
Morphological characterization and growth rate of RNT and RH cells Phase-contrast microscopy and H&E staining of cultured RNT **A., C**. and RH **B., D**. cells. Magnification 20x. For the experimental procedure followed to obtain the cell lines, see Materials and Methods. **E**. The growth rate of the two cell lines in adherent conditions, in optimal (10% serum) and suboptimal (2% serum) growing conditions, was measured at the indicated times. Cells were fixed and stained with crystal violet; the dye retained by the cells was solubilized in 10% acetic acid and the Optical Density (570nm) was measured. On the X axis is shown the fold change increase of cell number, compared to time zero. ** P<0.01; ****P<0.0001.

RNT cells exhibit a clear hepatocyte morphology, as they show a typical polygonal architecture and big rounded nuclei; these cells are serum-dependent and show contact inhibition when growing in monolayer (Figure 1A, 1C, 1E). On the opposite, a more elongated morphology (fibroblast-like) characterizes RH cells (Figure 1B, 1D), that are able to proliferate under suboptimal culture conditions (low serum, Figure 1E), losing cell-cell contact inhibition and continuing to divide and forming multilayered foci.

**Figure 2:**
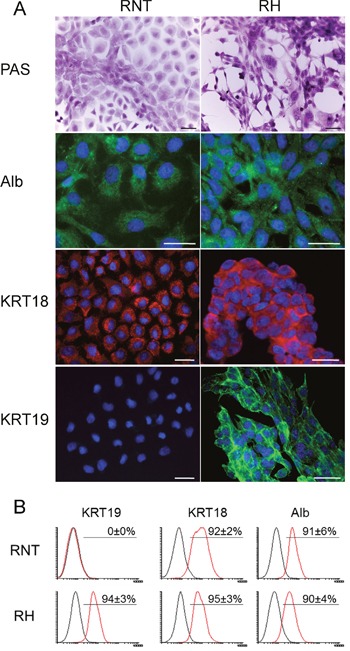
Profiling of typical hepatocyte markers in RNT and RH cells **A**. Microphotographs showing positivity for glycogen (PAS staining); **A., B**. Staining for Albumin (Alb), Cytokeratin 18 (KRT18) and Cytokeratin 19 (KRT19) was assessed by IF (A) and flow cytometry **B**. analysis in both cell lines. Bars = 25 μm.

**Figure 3:**
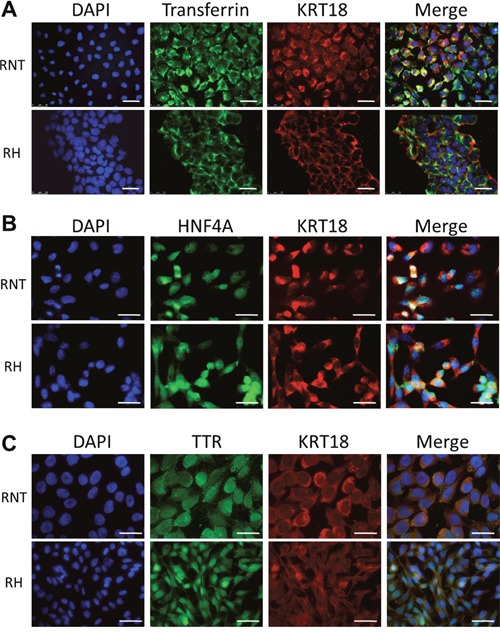
Expression of transferrin, hepatocyte nuclear factor 4 alpha and transthyretin in RNT and RH cells Co-expression of Transferrin (green) **A**., hepatocyte nuclear factor 4 alpha (HNF4A) (green) **B**., transthyretin (TTR) (green) **C**. and cytokeratin 18 (KRT18; red) in RNT and RH cells. Nuclei were stained with DAPI. Merge of the different stainings is shown in the last column on the right. Bars = 25 μm.

**Figure 4:**
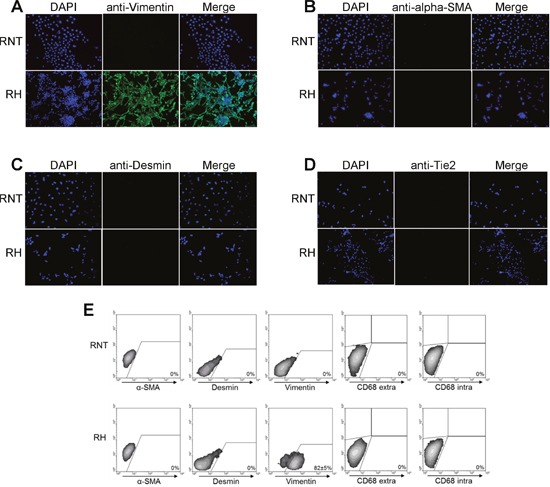
Panel of non-hepatocyte markers in RNT and RH cells Immunofluorescent staining for Vimentin **A**., alpha-SMA **B**., Desmin **C**. and Tie-2 **D**. Magnification 20X. **E**. Flow cytometry analysis of the expression of alpha-SMA, Desmin, Vimentin and the intra/extracellular macrophage marker CD68.

Next, we further characterized RNT and RH cells for the expression of hepatocyte and non-hepatocyte markers. Both cell lines were positive for glycogen (as shown by PAS staining), a classical marker of hepatocyte function (Figure 2A). Immunofluorescence and flow cytometry analysis showed that both cell types were also positive for canonical hepatocyte cell markers, such as albumin (Alb, >90%) and cytokeratin-18 (KRT18, >95%) (Figure 2A, 2B), Moreover, immunofluorescence for transthyretin (TTR), hepatocyte nuclear factor 4-alpha (HNF4A) and transferrin further confirmed the hepatocytic nature of the cells (Figure 3A-3C and Supplementary Figure 2). Performing the analysis for non-hepatocyte markers, we found that only the RH cell line displayed positivity for cytokeratin-19 (KRT19), a typical marker of bile ductular cells and of the so-called oval cells, emerging in pathological conditions [8, 9], including pre- and neoplastic stages (Figure 2A, 2B) [10, 11]. Interestingly, the intermediate filament vimentin - a marker of mesenchymal origin/feature [12] and involved in epithelial-mesenchimal transition (EMT) - was strongly expressed in RH cells, while it was almost completely absent in RNT cells (Figure 4A).

To rule out the presence of non-parenchymal cells, which could have grown together with hepatocytes, we performed immunofluorescent staining for alpha-smooth muscle actin (SMA) (Figure 4B), a marker of activated hepatic stellate cells [13], and for desmin (Figure 4C), a typical intermediate filament in cardiac, skeletal and smooth muscles [13]. The results showed that neither RH, nor RNT exhibited positivity for these non-hepatocyte markers. Lack of desmin, α-SMA and vimentin expression was confirmed by flow cytometry analysis (Figure 4E).Tie-2, the tyrosine kinase receptor for angiopoietin 1 [14, 15], is expressed almost exclusively in endothelial cells, in a fraction of monocytes and hematopoietic stem cells [14, 15]. Immunofluorescent staining for this receptor showed that neither RNT nor RH cells did express it (Figure 4D). Additionally, flow cytometry analysis revealed no expression in either cell line of intracellular and extracellular CD68, a macrophage marker (Figure 4E).

**Figure 5:**
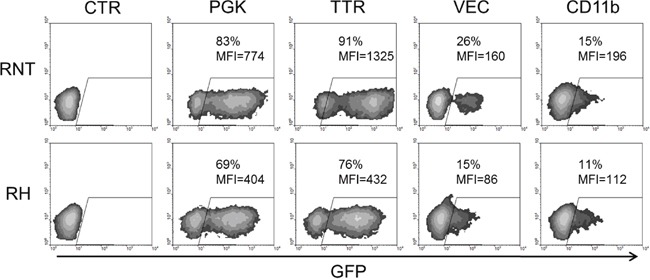
Lentiviral transduction and FACS analysis for promoter-characterization RNT and RH cells were transduced with four different Lentiviruses (LVs) containing the GFP transgene under the control of ubiquitous (PGK) or cell-specific promoters (TTR, hepatocyte-specific; VEC, endothelial-specific; CD11b, myeloid cells-specific) at MOI 1. GFP expression was evaluated by FACS analysis 72 hours after transduction. GFP expression driven by the hepatocyte-specific TTR promoter in both cell types is comparable to the expression driven by the ubiquitous PGK promoter, while VEC and CD11b promoters were less active in both cell types, confirming the hepatocyte phenotype of these cells. MFI = mean fluorescent intensity.

Moreover, to prove the hepatocytic nature of the two cell lines, we transduced them with lentiviral vectors expressing GFP under the transcriptional regulation of either hepatocyte-specific promoter or ubiquitous, endothelial- and myeloid-specific promoters. Since transthyretin (TTR) is a hepatocyte secreted protein, its promoter specifically drives transgene expression in hepatocytes [16]. At Multiplicity of Infection (MOI) of 1, in cultured RH and RNT cells, the percentage of GFP positive cells was similar to those transduced with a lentiviral vector containing a ubiquitously expressed promoter, the phosphoglycerate kinase 1 (PGK1) promoter (respectively 76% and 91% for TTR, 69% and 83% for PGK1), confirming the hepatocytic origin of these cells (Figure 5). On the contrary, promoters specific for endothelial and myeloid cells showed low levels of GFP expression (15% and 26% for VEC and 11% and 15% for CD11b), further demonstrating that the isolated cells were hepatocyte-derived. At the 0.1 MOI the differences were even more pronounced, in line with the demonstration that these cells are indeed hepatocytes (Supplementary Figure 3A). To validate the specificity of expression of the different described Lentiviruses, we transduced hepatocytic (C1C7), endothelial (MS1) and monocytic (U937) cell lines as controls (Supplementary Figure 3B).

All together, these results demonstrate the hepatocytic nature of cultured RNT and RH cells and rule out a possible contamination by endothelial, macrophages or stellate cells.

### RH but not RNT cells express tumor stem cell markers

Recently, putative liver tumor-initiating cells (T-ICs) have been identified by several cell surface antigens, such as CD90.1, EpCAM and CD24 [17–20]. T-ICs have been suggested to be critical for the maintenance, self-renewal, differentiation, and metastasis of tumors and to significantly impact patients’ clinical outcome [21]. Therefore, we investigated by flow cytometry the presence of T-ICs-like cells in both rat cell lines.

**Figure 6:**
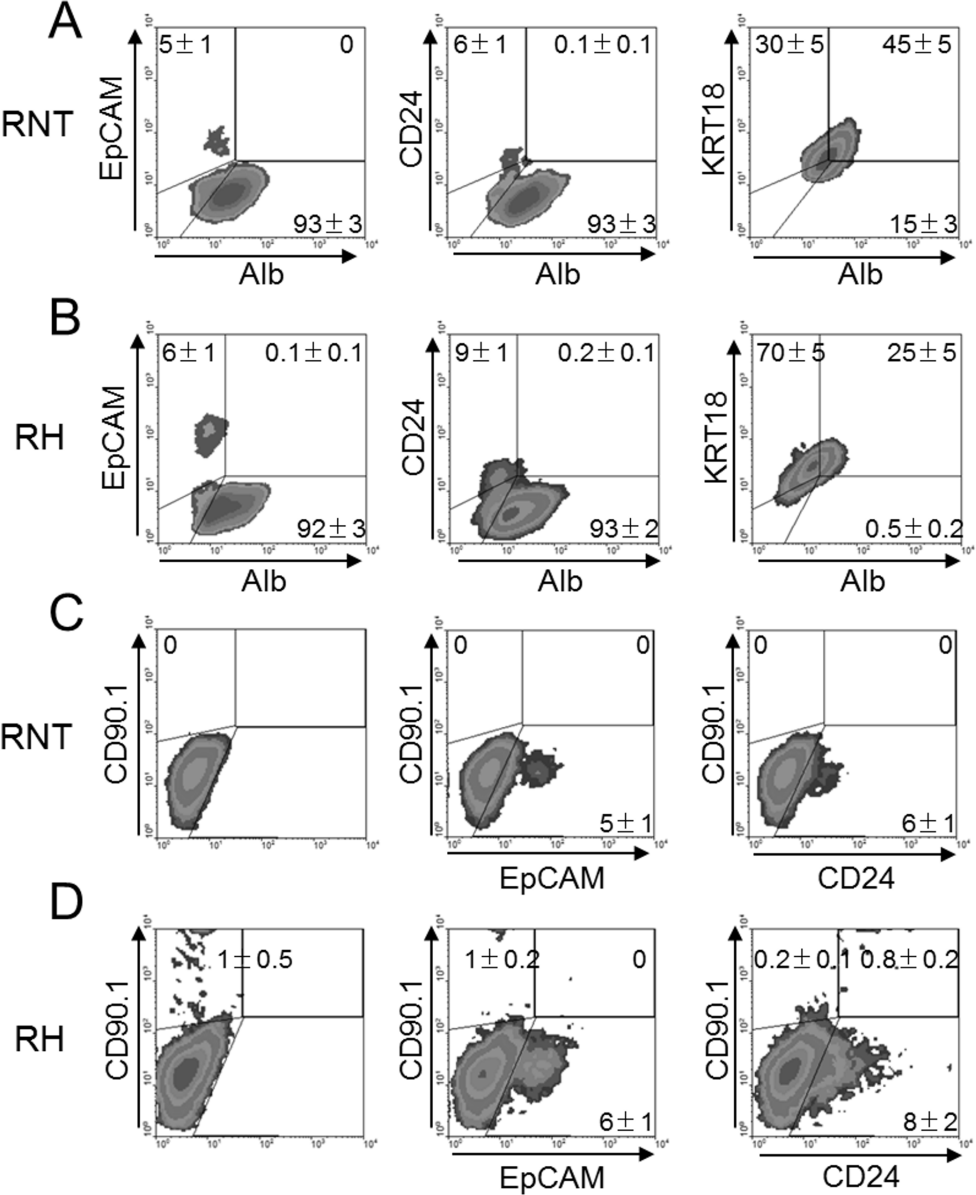
Identification of stem cell-like markers in RNT and RH cells RNT **A and C**. and RH **B and D**. cells were analyzed by flow cytometry for the expression of mature hepatocyte markers (Alb and KRT18), hepatocyte precursor markers (EpCAM and CD24) or the cancer stem cell marker (CD90.1). Numbers indicate the mean of at least 5 different analysis performed ± SD.

As shown in Figure 6A–6D, a small percentage of these two cell lines expressed EpCAM (RNT 5% and RH 6%), CD24 (RNT 6% and RH 10%) and CD90.1 (RNT 0% AND RH 1%). Interestingly, while EpCAM^-^ and CD24^-^ cells co-expressed albumin and cytokeratin 18, EpCAM^+^ and CD24^+^ cells were negative for these differentiation markers, further supporting their stem-like nature. Moreover, only RH cells were double positive for CD90.1/CD24, (Figure 6D), as CD90.1 was not detected in the immortalized-non tumorigenic RNT cell line (Figure 6C). These findings show that only RH cells contain tumor stem-like cells, as defined by the double expression of CD90.1 and CD24, in agreement with the results obtained from human samples [21].

**Figure 7:**
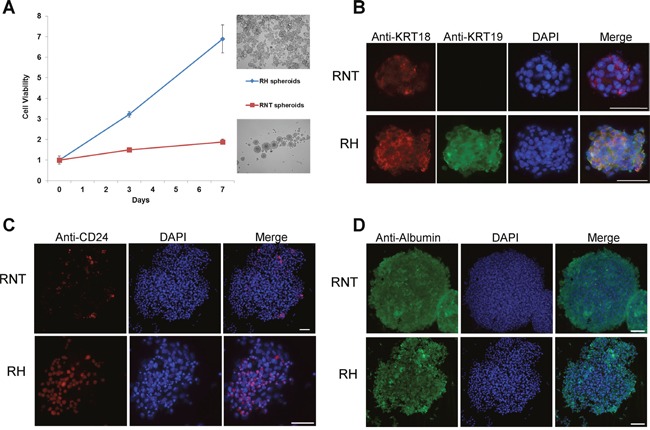
Generation of RNT and RH spheroids **A**. The two cell lines were grown in stem medium and low attachment conditions; cell viability was measured at the indicated times. Pictures in the right part of the graph represent RH (upper figure) and RNT spheroids (lower figure) at day 7 (magnification 10X). The X axis shows the fold change increase in cell viability, compared to time zero. Immunofluorescence showing expression of hepatocytes markers KRT18 **B**. and Alb **D**. in spheroids of both cell types; on the contrary, KRT19 is present only in RH spheroids **(B)**. A small percentage of RH and RNT spheroids are positive for CD24 **C**. the number of CD24^+^ spheroids is higher in RH cells **(C)**. Scale bar = 25 μm.

To further characterize the two cell lines, we analyzed their growth ability in 3D cultures. We thus generated spheroids and found that RH cells gave rise to more numerous spheroids than RNT cells (Figure 7A). Notably, both cell lines maintained in 3D culture the expression of hepato-specific markers, such as albumin and cytokeratin 18 (Figure 7B, 7D), and of the hepatic precursor marker CD24 (Figure 7C). In the RNT cells CD24 was less represented and the staining for KRT19 (Figure 7B, 7C) was negative confirming the result obtained in the 2D culture of RNT cells (Figure 2A–2B).

**Figure 8:**
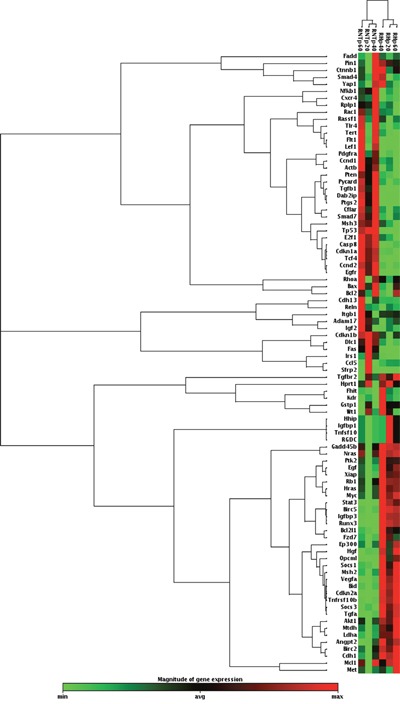
Hierarchical clustering of 84 genes in RNT and RH cells Each row represents the expression profile of a gene. Only mRNAs whose expression was dysregulated at least by 2-fold were considered. Red and green colors represent higher or lower expression levels of the mRNA (median-centered), respectively.

To evaluate the gene expression profile of these two cell types we used the Rat Liver Cancer RT^2^ Profiler PCR Array and found that 44 out of the 84 genes present in the Array were differentially regulated between RH and RNT cells (Threshold = fold change +/−2) (Supplementary Table 1). As shown in Figure 8, while the Heat Map displayed only minimal differences in each cell type at different culturing passages (20, 40, 60), the dendrogram clearly separated RH from RNT. Furthermore, by comparing the expression profile of RH and RNT with normal livers and primary HCCs, we found that RNT clustered together with control livers, while RH were much closer to the HCC sub-cluster (Supplementary Figure 4). Quantitative RT-PCR validation performed on randomly selected genes (*Birc5, Igfbp1, Myc, Tert, Cnnd1* and *Met)* confirmed the microarray expression data for all the examined genes (Supplementary Figure 5).

DAVID Functional Analysis of genes altered in RH cells and HCC *vs*. their respective controls revealed that most of the dysregulated genes are involved in Ubiquitin conjugation, Apoptosis, Phosphoprotein, Glycoprotein and Signal (Supplementary Figure 6A). Pathway analysis also underlined common modifications between RH cells and HCCs (Pathways in Cancer, MicroRNA in cancer, Hepatitis B, PI3K-AKT-signaling pathway) (Supplementary Figure 6B).

Collectively, these results show that RH and RNT cells not only exhibit a distinct expression profile, but they also maintain features displayed *in vivo* by transformed and normal hepatocytes, respectively.

### RH cells, but not RNT, are endowed with a transformed/tumorigenic potential

Cell immortalization can be accompanied by transformation. Thus, for both the cell lines, we assessed the transformed/tumorigenic potential typically associated *in vitro* with the acquisition of anchorage-independent growth ability and *in vivo* with the capacity to generate tumors when grafted.

**Figure 9:**
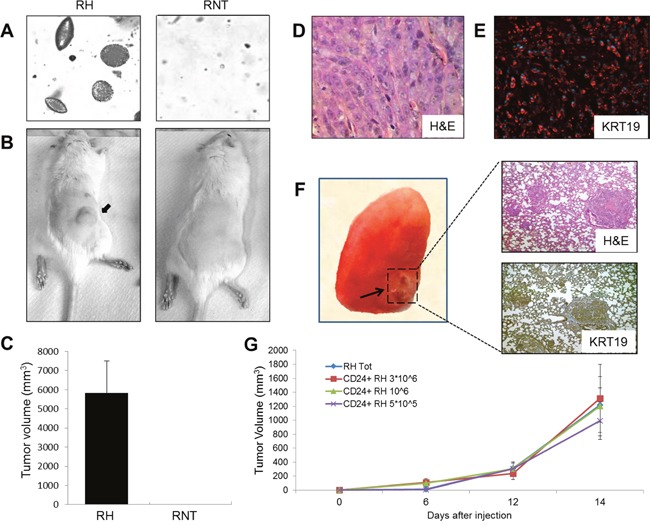
Biological assays and tumorigenesis **A**. The ability to grow in anchorage-independent conditions was assessed by soft agar assay. Colonies were photographed after two weeks. **B**. Representative photographs of syngeneic rats injected with RH (left) or RNT (right) cells. RH and RNT cells were inoculated into the posterior flank of syngeneic rats (1.5*10^6^ cells/injection); **C**. Graph showing the tumor volume of rats, 19 days after injection with RH or RNT. The results represent the mean ± SD of 5 animals/group; **D**. Microphotograph showing H&E staining of a HCC developed 19 days after injection of RH cells; **E**. Microphotograph illustrating a strong positivity of KRT19 in the same HCC; **F**. (Left) Macroscopic photograph of a lung from a rat injected with RH cells and killed 30 days after grafting. A metastasis is shown by the arrow. (Right) Microphotographs of the metastasis shown in F, stained for H&E (top) and KRT19 (bottom; Magnification 10x); **G**. Unselected (Tot) and CD24^+^ RH cells were inoculated into the posterior flank of NOD-SCID γNull mice (10^7^ cells/injection for unselected RH cells and 5×10^5^, 1 × 10^6^ and 3 x10^6^ for CD24^+^ RH cells). The graph shows tumor growth in mice up to 14 days after injection. The results represent the mean ± SD of 5 animals/group.

When plated in soft agar, RH cells were able to form numerous and large colonies within seven days, while RNT cells did not, even after 3 weeks. (Figure 9A). Moreover, while RH cells were able to form tumor masses in 30 days, when inoculated into the posterior flank of syngeneic rats, RNT cells were completely devoid of tumorigenic ability (Figure 9B, 9C); these tumors showed morphological features of HCC (Figure 9D) and were strongly positive for KRT19 (Figure 9E), thus maintaining the same features of the cells grown *in vitro*.

As RH cells display EMT features, we wondered if they were able to form metastases. Indeed, one month after grafting, we observed the appearance of lung macro-metastases (Figure 9F). H&E and immunohistochemical staining on serial sections of lung metastases showed the maintenance of primary HCC morphology and KRT19 positivity. As expected, RNT cells were unable to originate metastases up to 3 months after grafting (data not shown).

To investigate whether CD24^+^ stem-like cells are involved in tumor formation, 5×10^5^ to 3×10^6^ CD24^+^ and CD24^-^ RH and RNT cells were injected s.c. into the posterior flank of NOD-SCID yNull mice (n=5), immediately after isolation. While both CD24 positive and negative RNT cells did not give rise to tumors up to 3 months, CD24^+^ RH cells generated tumors with a similar size within 14 days in all tested conditions (Figure 9G). As controls we injected 10^7^ unselected RH and RNT cells and only the RH cells formed tumors similar in size to those observed with CD24 positive RH cells. Interestingly, CD24^-^ RH cells did not give rise to tumors, confirming that tumorigenic cells are in the CD24^+^ fraction (data not shown). Of note, we found that among the CD24^+^ RH cells, 7±2% were also CD90.1^+^, suggesting that this subpopulation could be responsible for the tumorigenic potential of RH cells. In agreement, the CD24^+^ RNT cells did not contain any CD90.1^+^ cell (Supplementary Figure 7).

### RNT/RH cells as a tool to investigate the molecular mechanisms of hepatocarcinogenesis

The two cell lines we have established represent two different stages of the hepatocarcinogenic process. To provide a proof of concept that they can be used to study the role of candidate molecules in HCC progression, we focused our interest on NRF2. NRF2 is an integrated redox sensitive signaling system that regulates 1%-10% of human genes and is negatively controlled by the ubiquitin ligase KEAP1, which promotes NRF2 proteasome-mediated degradation [22, 23].

**Figure 10:**
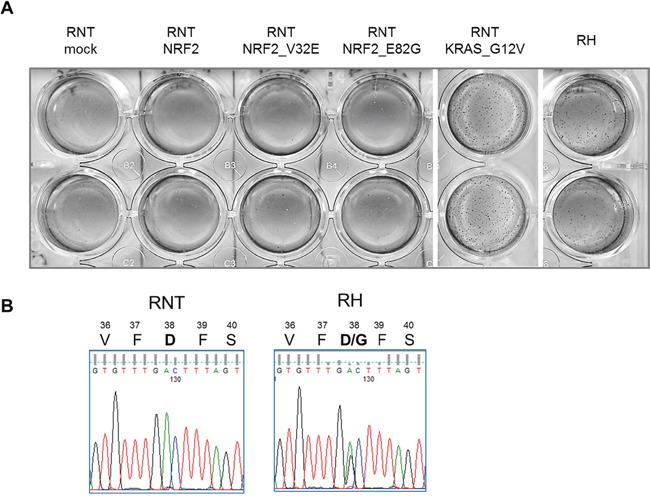
NRF2 transduction is not sufficient to transform RNT cells **A**. RNT cells were transduced with the indicated constructs (mock vector, wild type NRF2, NRF2 constructs bearing activating mutations, activated KRAS). The ability to grow in anchorage-independent conditions was assessed by soft agar assay. Colonies were photographed after two weeks. RH cells were used as positive controls for the growth in soft agar. **B**. Pherograms showing the presence of a NRF2 activating mutation (D38G) in RH but not in RNT cells.

As previously published [24], the NRF2 pathway is already activated in early preneoplastic lesions and along tumor progression. NRF2 silencing in RH cells inhibits their tumorigenic ability, both *in vitro* and *in vivo* [25], demonstrating that activation of this pathway is necessary to sustain the malignant phenotype. To investigate if NRF2 activation is “sufficient” to confer transforming ability to RNT cells, we transduced them with a lentiviral vector containing NRF2 cDNA and evaluated their *in vitro* transforming ability. As shown in Figure 10A, over-expression of NRF2 was not sufficient to confer to the cells the ability to grow in an anchorage-independent manner.

We previously showed that *Nrf2/Keap1* mutations are present in 59.3% of HCCs developed in the R-H model [25]. Interestingly, while RNT cells are wild type for NRF2, RH cells display an activating mutation (Figure 10B). Therefore, to further stress the system, we transduced RNT cells with constitutively activated forms of this gene, as consequence of activating mutations (Supplementary Figure 8). Transduction of RNT cells with two different NRF2 mutated forms did not confer them *in vitro* tumorigenic ability. However, RNT cells could be transformed by transduction of a well-known oncogene, such as the active (G12V) KRAS (Figure 10A).

All together, these experiments show that NRF2 activation is necessary to sustain the tumorigenic status but is not sufficient *per se* to induce it.

## DISCUSSION

In the present paper, we report the *in vitro* establishment and the functional characterization of two novel rat immortalized hepatocytic cell lines: RH and RNT. Both cell lines were obtained by perfusion of rat livers exposed to the Resistant-Hepatocyte protocol, in the presence or in the absence of carcinogenic treatment, and could be maintained in culture for more than 50 passages, without signs of senescence (data not shown).

Both cell lines display features typical of hepatocytes as they are glycogen-positive, produce albumin and express KRT18, and are not contaminated by other cell types usually present in the liver, such as macrophages, stellate cells, and endothelial cells. However, obvious differences exist between them, as RNT cells display a typical hepatocytic morphology, while RH cells show a fibroblastoid shape, with signs of EMT. EMT is a critical event in the induction of cell motility and invasion, both in physiological conditions - like wound healing or development - and in malignant transformation – when tumor cells acquire invasive/metastatic properties. Indeed, vimentin - an intermediate filament protein characteristically upregulated in cells undergoing EMT [12, 26, 27] - was only weakly expressed in RNT cells, while it was strongly up-regulated in RH cells, in agreement with their fibroblastoid shape and increased metastatic ability.

The performed gene expression profiling showed not only that many of the evaluated genes were differentially expressed, but also that RNT cells display a profile similar to normal livers while RH are very similar to HCCs.

Another relevant difference concerns the expression of KRT19 that was observed only in RH tumorigenic cells. In normal liver, hepatocytes express KRT8 and KRT18, whereas biliary epithelial cells express KRT7 and KRT19 [8, 9]. However, the presence of KRT19^+^ hepatocytes has been demonstrated in a subset of human HCCs characterized by the worst prognosis among all HCC subclasses, suggesting that KRT19 is a negative prognostic marker [11, 28]. Moreover, in the Resistant-Hepatocyte model, although KRT19^+^ lesions represent a minority of the total preneoplastic lesions, most HCCs are KRT19^+^, further suggesting that KRT19^+^ preneoplastic hepatocytes preferentially progress to malignancy [10, 24]. Furthermore, KRT19^+^ HCCs show significantly increased EMT features and expression of invasion-related molecules, suggesting that they are endowed with higher invasive ability, compared to KRT19^-^ HCCs [28].

Another important difference between the two cell lines is the presence of tumor stem-like cells only in the RH cell line. We have investigated three markers that have been associated to T-ICs, namely CD90.1, CD24 and EpCAM [17–20]. The CD90.1 (Thy-1) antigen is expressed in bone-marrow derived stem cells and hepatic stem/progenitor cells (both in adult and fetal livers, but not in adult hepatocytes)[29]. In the liver, CD90.1 expression was found preferably in poorly differentiated HCCs and associated with a poor prognosis [30]. Moreover, only CD90.1^+^ cells obtained from HCCs displayed tumorigenic and metastatic capacity when injected into immunodeficient mice [31]. CD24, which is overexpressed in various human malignancies [32, 33], was expressed at higher level in RH than in RNT cells. More interestingly, the CD90.1 marker was present only in RH cells, where it is co-expressed within the CD24^+^ population. Actually, both markers were reported to be involved in CSC differentiation in HCC [34]. Interestingly, only the CD24^+^ RH population (containing also CD90.1^+^ cells) was endowed with tumorigenic ability, while neither CD24^-^ RH cells nor CD24^+^ RNT cells (that do not display CD90.1^+^), gave rise to tumors after subcutaneous implantation in mice.

A critical difference between the two cell lines is that only RH cells displayed the typical behavior of malignant transformed cells, as *in vitro* they grow in anchorage-independent manner and do not show contact inhibition, and *in vivo* are strongly tumorigenic and metastatic. Interestingly, RNT cells are immortalized but not tumorigenic. Thus, they represent a critical “normal” counterpart of RH transformed cells. As these two cell lines epitomize two steps in the natural history of tumor development, they can therefore be used to study the molecular mechanisms underlying tumor progression. Indeed, we have shown that NRF2 silencing in RH cells reverted their phenotype toward that of RNT cells, demonstrating that activation of the NRF2 pathway is required to maintain the transformed phenotype [25]. However, exogenous expression of NRF2 - or even of activated forms of this gene - did not promote the progression toward a transformed phenotype. On the other hand RNT cells could be transformed by transduction with an oncogenic form of KRAS, thus proving their susceptibility to become tumorigenic. All together, these results suggest that NRF2 constitutive activation is not sufficient to promote the transformation of the immortalized liver cells. The analysis of differentially expressed and mutated genes in RH vs. RNT cells can thus help in the identification of the genes that complement NRF2 in promoting transformation. Exogenous expression/silencing of selected genes will allow to experimentally prove the results obtained *in silico*.

In conclusion, the two cell lines here described represent a useful tool for investigating the molecular changes underlying hepatocyte transformation and to experimentally demonstrate their role in HCC development.

## MATERIALS AND METHODS

### Animals and treatment

Guidelines for Care and Use of Laboratory Animals were followed during the investigation. All animal procedures were approved by the Ethical Commission of the University of Cagliari and the Italian Ministry of Health. Male Fischer F-344 rats (100-125 g) purchased from Charles River (Milan, Italy) and NOD.*Cg-PrkdcscidIl2rgtm1Wjl/SzJ mice* (γNull) were originally by Jackson Laboratories (Bar Harbor, Maine, USA). Animals were kept on a laboratory diet (Ditta Mucedola, Milan, Italy) and given food and water *ad libitum* with a 12-hour light/dark daily cycle.

HCC was induced according to the Resistant Hepatocytes (R-H) model [7]. Rats were injected intraperitoneally with the chemical carcinogen diethylnitrosamine (DENA, Sigma, MO) at a dose of 150 mg/kg body weight. After a 2-week recovery, rats were fed a diet containing 0.02% 2-acetylaminofluorene (2-AAF, Sigma, MO) for 1 week followed by a two-thirds partial hepatectomy (2/3 PH), and an additional week of 2-acetylaminofluorene diet. The animals were then returned to the basal diet and euthanized at 14 months (Supplementary Figure 1). Rats exposed to 2-AAF and 2/3 PH, but in the absence of carcinogen, were used as controls.

### Cell isolation and culturing

RNT (Rat Not Tumorigenic) and RH (Resistant Hepatocytes) cells were obtained from rats treated with 2-AAF + PH (rats with no tumors) or DENA + 2-AAF + PH (tumor bearing rats), respectively. Livers were perfused at 10 ml/min via portal vein for 5 minutes with Leffert's buffer (at 37°C) containing 1.9 mg/ml EGTA, for 2 minutes with buffer lacking EGTA, and for 10-15 minutes with buffer containing 0.03% (w/v) collagenase (Worthington Biochemical Corp.) and 5 mM CaCl_2_.2H_2_O, as described [35], with modification for rat liver. The perfusion buffer contained 10 mmol/l HEPES, 3 mmol/l KCl, 130 mmol/l NaCl, 1 mmol/l NaH_2_PO_4_.H_2_O, and 10 mmol/l d-glucose, pH 7.4). The livers were dissociated in Leffert's buffer, and cells were passed through Dacron fabric with 80-μm pores and centrifuged under 50 *g* for 5 minutes to recover hepatocytes. For livers containing tumors, digestion continued in the plastic dish for additional 10-20 min in the presence of collagenase: after incubation, tumor masses present in the remaining parenchyma were mechanically disrupted. Cells were recovered and washed several times in serum-free medium; then hepatocytes were maintained in petri dishes coated with rat tail collagen 0,2%, in 10% FBS DMEM.

Murine peritoneal macrophages were collected by intraperitoneal injection of RPMI-1640 and maintained in 5% FBS RPMI-1640.

Cultured RNT and RH cells were maintained in 10% FBS RPMI with P/S (100U/ml Penicillin, 100mg/l Streptamicin), and L-Glutamine (2mM). C1C7 (murine HCC cell line), MS1 (murine endothelial cell line), U937 (human macrophage cell line) and HTC (rat hepatoma cell line) were maintained in 10% FBS DMEM.

### RNT and RH spheroids

To obtain spheroids from RNT and RH cells, cells were seeded in low-attachment 24-well plates (10^4^ cells/well) in stem medium conditions (0% FBS DMEM-F12 medium, supplemented with EGF, bFGF, insulin and B-27 supplement). After the formation of spheroids, they were seeded in low-attachment 96-well plates and cell viability was evaluated at days 0, 3 and 7 after seeding, using CellTiterGlo assay (Promega).

### Periodic acid-schiff (PAS) stain for glycogen

Culture dishes containing cells were fixed in ethanol - acetic acid (99:1) for 10 minutes at 4°C, incubated with Schiff reagent for 5 minutes and washed twice with periodic acid (Carlo Erba).

### Immunofluorescence (IF) analysis

Cells were seeded on collagen-coated coverslips for 48h, subsequently rinsed with PBS and subjected to immunofluorescence. RNT and RH spheroids were prepared by cyto-spin at 1000 rpm for 5 min. Cells were fixed with 4% paraformaldehyde (Sigma Aldrich) or with methanol (Sigma-Aldrich) at room temperature and permeabilized with PBS containing 0.1% Triton X-100 (Sigma-Aldrich). After blocking with 5% goat serum (Sigma Aldrich), cells were subjected to immunofluorescence staining with primary antibodies diluted in PBS containing 1% BSA, 0.1% Triton X-100 and 2% goat serum, as listed in Supplementary Table 2.

Cells were finally incubated with either secondary anti-mouse, anti-rabbit AlexaFluor 488-conjugated or AlexaFluor 546-conjugated antibodies (dilution 1:500, Invitrogen, Carlsbad, CA) for 1 h in PBS containing 1% BSA and 0.1% Triton X-100. Nuclei were stained with DAPI (Sigma Aldrich). As a control, staining of RNT and RH was performed with the secondary antibody alone (Supplementary Figure 9). Cells were examined by fluorescence microscopy (Olympus America Inc, Center Valley, PA). Images were acquired as sets of color-images and prepared using Photopaint and Photoshop software.

### Cytofluorimetric analysis

To analyze extracellular markers, cells were re-suspended in staining buffer (PBS, 1% FBS, 0.1% NaN_3_) followed by incubation with specific antibodies for 30 minutes on ice. For intracellular markers, cells were re-suspended in staining buffer containing 1% paraformaldehyde for 5 minutes, incubated with Perm wash buffer (BD Biosciences, San Diego, CA) for 5 minutes, followed by incubation with primary antibody in Perm/Wash buffer for 30 minutes on ice. Finally, cells were washed twice in Perm/Wash buffer (BD Biosciences) and re-suspended in staining buffer. Specific antibodies used are listed in Supplementary Table 3.

### Lentiviral transduction

Four different lentiviral vectors expressing the green fluorescence protein (GFP) under the control of PGK (Phosphoglycerate kinase, ubiquitous), TTR (Transthyretin, hepatocyte specific), VEC (Vascular-Endothelial Cadherin, endothelial specific) and CD11b (Integrin alpha M, ITGAM, myeloid cells specific) promoters were used to transduce RH and RNT cells. As controls we used the following cell lines: C1C7 murine hepatocytes, MS1 murine endothelial cells, U937 human monocytes. Cells were transduced with a multiplicity of infection (MOI) of 1 and 0.1. Expression level and stability of the GFP expression was evaluated by flow cytometry 72 h after transduction.

RNT cells were stably transduced with an empty lentiviral vector (mock), with an NRF2 lentiviral construct (217EX-T3128-Lv157; GeneCopoeia, Rockville, MD), with the mutant forms of NRF2 (V32E and E82G), obtained using the QuikChange II XL Site-Directed Mutagenesis Kit (Agilent Technologies), and with a lentiviral construct expressing the activated form of KRAS G12V.

### CD24^+^ cells isolation

For CD24^+^ cells isolation, RH and RNT cells were first incubated for 20 min at 4°C with anti-rat CD24 PE-conjugated antibody (Miltenyi Biotec) followed by a second incubation with anti-PE Microbeads (Miltenyi Biotec) for 20 min at 4°C and finally immunomagnetically separated using magnetic columns (Miltenyi Biotec). Cell purity was verified by flow cytometry. Freshly isolated cells were then counted and immediately injected s.c. into yNull mice.

### Cell growth

RNT and RH cells were seeded in 96-well plates (4000 cells/well) at two different concentrations of serum (2% and 10%). Cells were fixed in 11% glutaraldehyde and stained with crystal violet at days 1, 3 and 6 after seeding. The dye retained by the cells was then solubilized in 10% acetic acid and the Optical Density (570nm) was measured using a Multilabel Reader (PerkinElmer, Waltham, MT, USA).

For evaluation of anchorage-independent growth, 3000 cells/well were seeded in 10% FBS RPMI 0.5% soft agar and maintained in the presence of medium for 15 days. Grown colonies were visualized by staining with crystal violet and pictures acquired as sets of b/w images by microscope.

### RH and RNT expression profile by qRT-PCR

Total RNA from cultured RH and RNT at different culturing passages and from normal rat livers and HCCs was first extracted by using TRIzol® reagent (Invitrogen) and further purified using the RNeasy Micro Kit (Qiagen). cDNA was obtained from 1 μg of RNA using the RT^2^ First Strand Kit (Qiagen). Gene expression profile of RH, RNT, normal livers and tumors was analyzed by real-time qPCR using the Rat Liver Cancer RT^2^ Profiler™ PCR Array (Qiagen), that evaluates 84 genes. Analysis was performed using the Web-based PCR Array Data Analysis Software available at SAB website (www.SABiosciences.com/pcrdataanalysis.php). B2m was used as housekeeping gene. Only mRNAs whose expression was dysregulated by at least 2-fold compared to their controls were considered modified.

### QRT-PCR validation

Analysis of *Birc5, Igfbp1, Myc, Tert, Cnnd1* and *Met* expression was performed using specific TaqMan probes (Applied Biosystems); GAPDH was used as endogenous control. The results are the mean of three different samples/group.

### Pathway and functional analysis by means of the DAVID bioinformatics resources software

The functional enrichment analysis of the differentially expressed genes was performed with the Database for Annotation, Visualization and Integrated Discovery (DAVID), including gene ontology (GO) function analysis and Kyoto Encyclopedia of Genes and Genomes (KEGG) pathway analysis. Analysis of pathways and functions was based on the number of genes significantly dysregulated (fold difference cutoff + 2.0). In KEGG pathway analysis, enriched pathways were identified according to the hypergeometric distribution with a P-value < 0.01.

### *In vivo* experiments

The rat *in vivo* experiments were performed by inoculating s.c.1.5 × 10^6^ cells (suspended in sterile PBS/Matrigel ratio 1:1) into the posterior flank of F344 male rats. For experiments performed in mice, 10^7^ unselected RH or RNT cells or 5×10^5^ – 3×10^6^ CD24^+^ or CD24^-^ RH or RNT cells were inoculated in the same manner in γNull mice. Tumor masses were measured with a caliper. Tumors were then resected and analyzed by H&E stain and IF. Lungs were surgically removed to histologically verify the presence of metastases.

## SUPPLEMENTARY MATERIALS FIGURES AND TABLES



## Abbreviations

R-H: resistant-hepatocyte model
DENA: diethylnitrosamine
AAF: acetylaminofluorene
PH: partial hepatectomy
RH cells: rat hepatocarcinoma cells
RNT cells: rat non-tumorigenic cells
HCC: hepatocellular carcinoma
KRT19: cytokeratin 19
TTR: transthyretin
PGK1: phosphoglycerate kinase 1
GFP: green fluorescent protein
HNF4A: hepatocyte nuclear factor 4-alpha
EMT: epithelial-mesenchimal transition
NRF2: nuclear factor (erythroid-derived 2)-like 2
VEC: Vascular-Endothelial Cadherin, endothelial specific
CD11b: Integrin alpha M, ITGAM, myeloid cells specific
