# Supplementary material for: A long term, non-tumorigenic rat hepatocyte cell line and its malignant counterpart, as tools to study hepatocarcinogenesis
Source: Oncotarget. 2017 Feb 1;8(9):15716–31. doi: 10.18632/oncotarget.14984 (PMC5362518; doi:10.18632/oncotarget.14984)
Supplement: Supplementary file 1 [file oncotarget-08-15716-s001.pdf]

A long term, non-tumorigenic rat hepatocyte cell line and its malignant counterpart, as tools to study hepatocarcinogenesis

SUPPLEMENTARY FIGURES AND TABLES

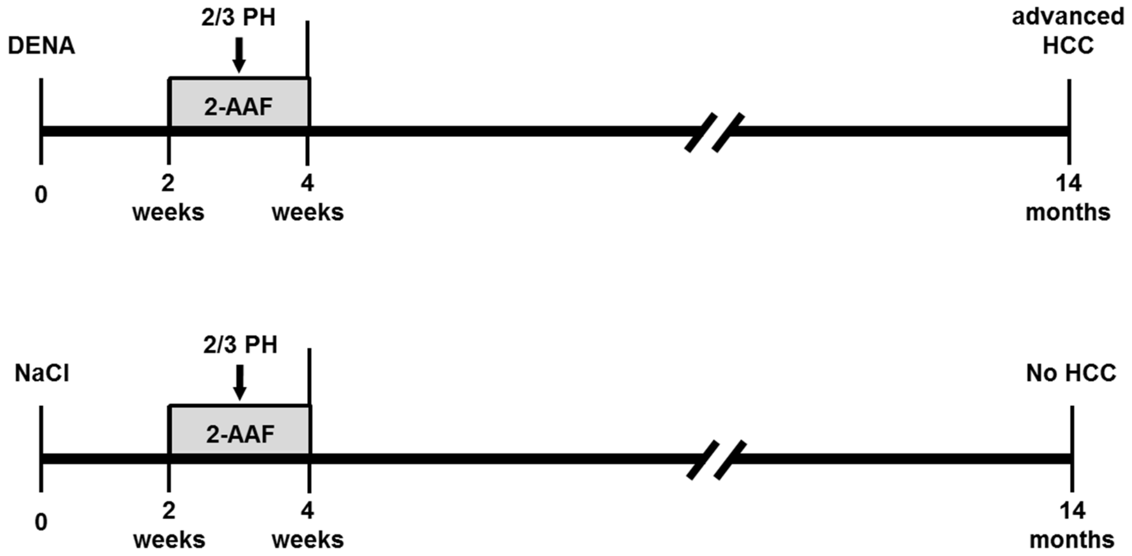

Supplementary Figure 1: Schematic representation of the experimental protocol. F-344 rats injected with a single intraperitoneal dose of diethylnitrosamine (DENA) or saline (NaCl) were subjected to the R-H protocol, consisting of a 2-week diet supplemented with 0.02% 2-acetylaminofluorene (2-AAF) and a two/thirds partial hepatectomy (2/3 PH). Rats were then switched to basal diet all throughout the experiment and killed 14 months after DENA administration.

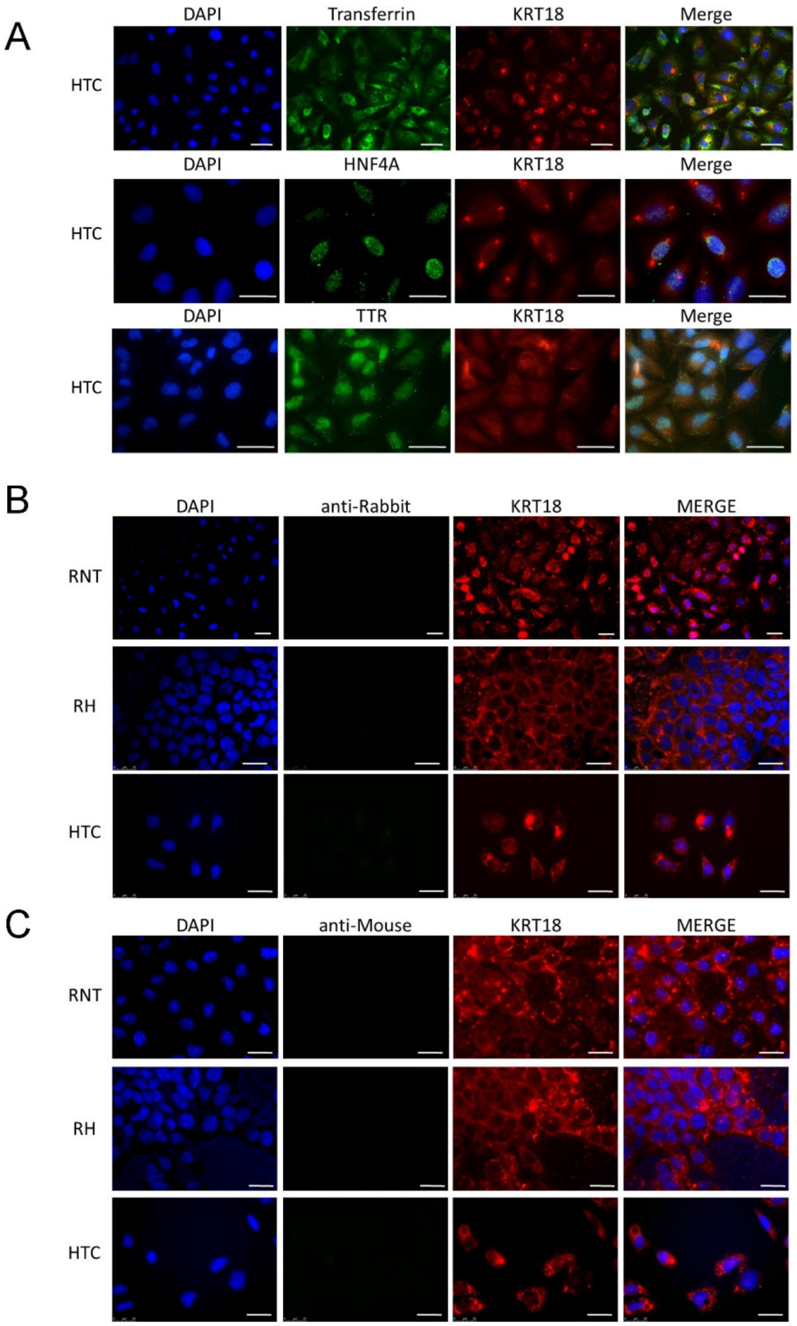

Supplementary Figure 2: Positive and negative controls for immunofluorescence on RNT, RH and HTC cells. A. HTC cells served as control cells for transferrin, HNF4A and TTR stainings. RH, RNT and HTC cells were stained with: i) DAPI for nuclear staining, ii) fluorochrome-conjugated secondary anti-rabbit **B.** or anti-mouse **C.** antibodies (Alexa Fluor® 488); iii) anti-KRT18 antibody. The right part shows the merged images of the different stainings. Bars = 25 µm.

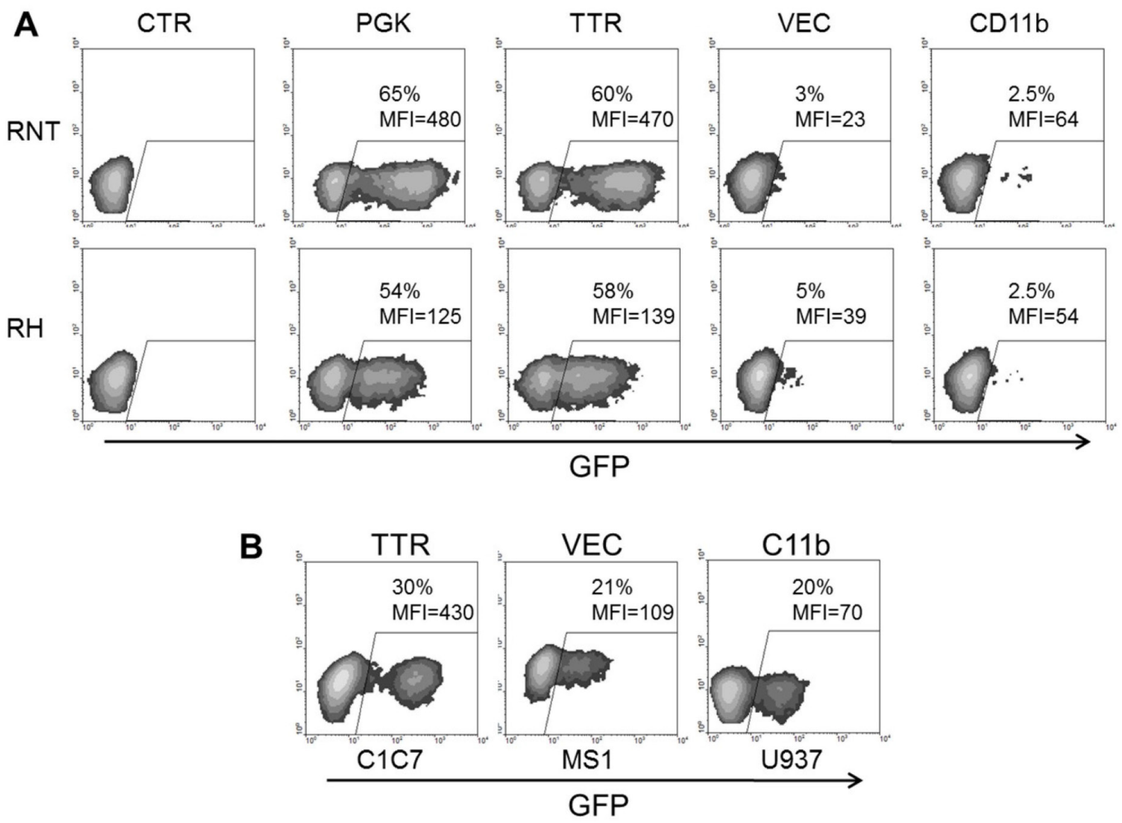

Supplementary Figure 3: Lentiviral Cell Transduction with cell-type specific promoters. Flow cytometry analysis shows RNT and RH cells **(A)** and control cells C1C7= murine hepatocytes; MS1= murine endothelial cells; U937= human monocytes). **(B)** transduced with four different LVs containing the GFP transgene under the control of ubiquitous (PGK) or cell-type specific promoters (TTR, hepatocyte-specific; VEC, endothelial-specific; CD11b, myeloid cells-specific) at MOI 0.1. After transduction, GFP expression was higher in cells transduced with LV containing the TTR promoter in both cell lines and comparable to the expression driven by the ubiquitous PGK promoter; GFP expression was very low in RH and RNT cells transduced with LVs containing VEC and CD11b promoters, confirming the hepatocyte phenotype of these cells. MFI = mean fluorescent intensity.

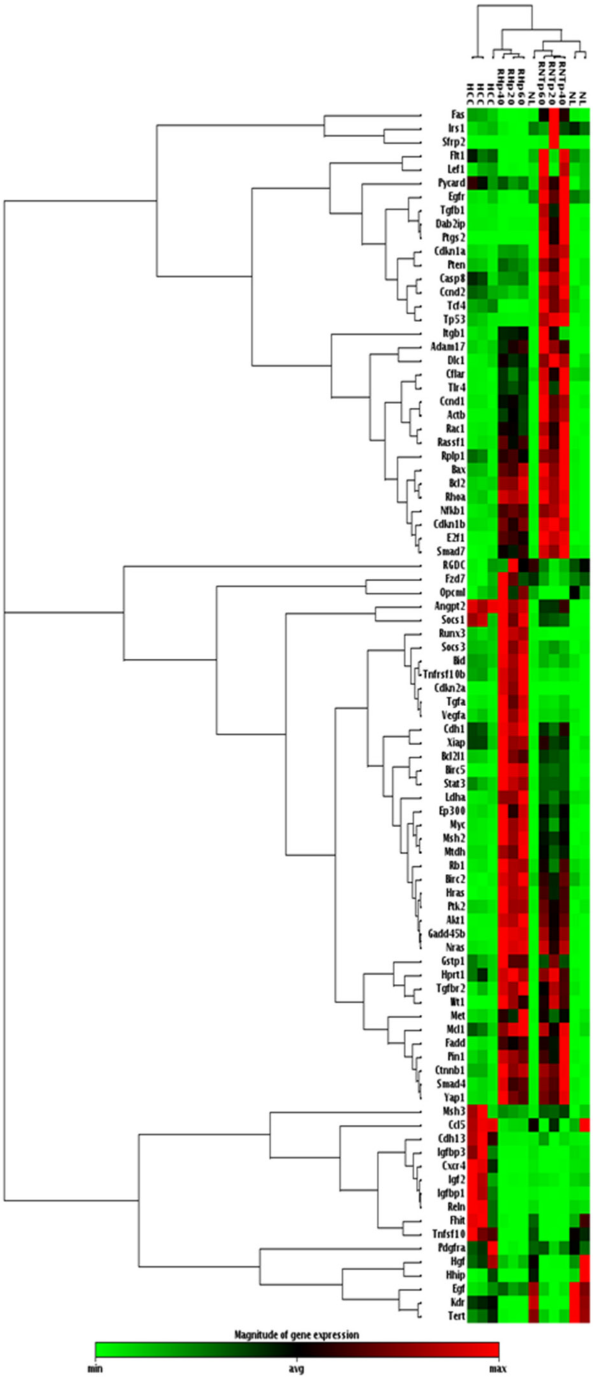

Supplementary Figure 4: Hierarchical clustering of 84 genes in RH and RNT cells, normal livers and HCCs generated 14 months after administration of DENA. Each row represents the expression profile of a gene. Only mRNAs whose expression was dysregulated at least by 2-fold were considered. Red and green colors represent higher or lower expression levels of the mRNA (median-centered), respectively.

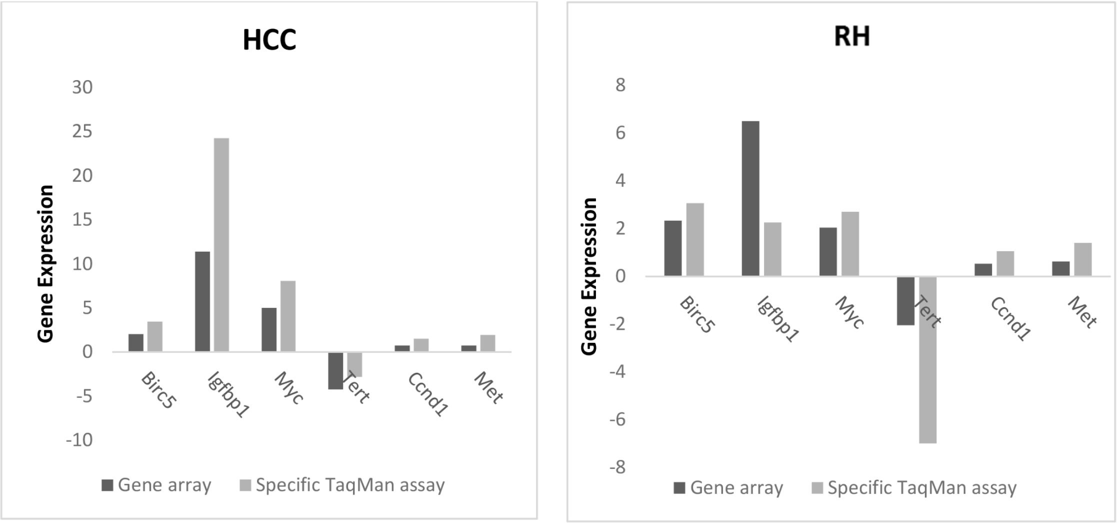

Supplementary Figure 5: QRT-PCR validation of randomly selected genes in rat HCC and RH cells. Gene expression is reported as log fold-change relative to control liver (left panel) and RNT (right panel), respectively.

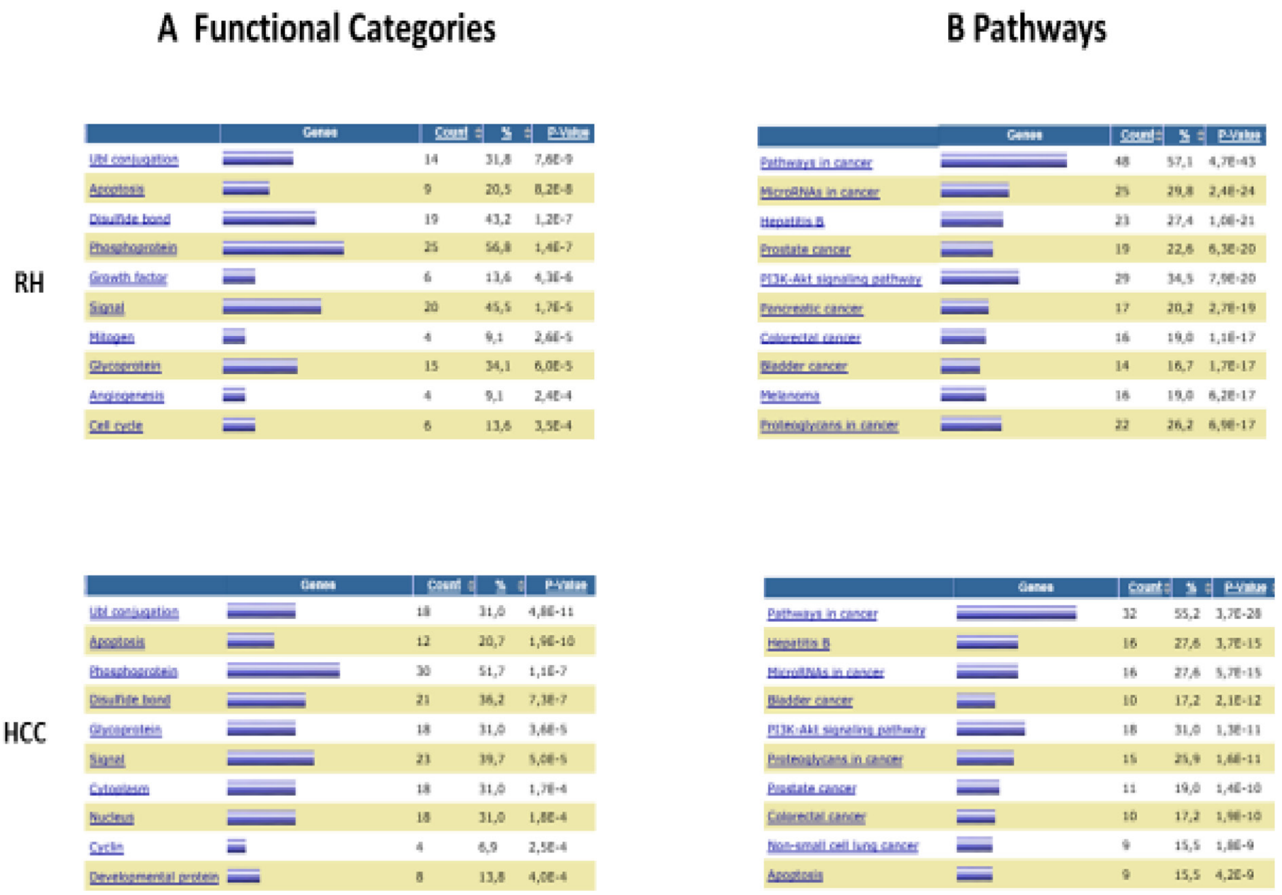

A Functional Categories

RH

	Genes	Count	%	P-Value
Ubl conjugation		14	31,8	7,6E-9
Apoptosis		9	20,5	8,2E-8
Disulfide bond		19	43,2	1,2E-7
Phosphoprotein		25	56,8	1,4E-7
Growth factor		6	13,6	4,3E-6
Signal		20	45,5	1,7E-5
Mitogen		4	9,1	2,6E-5
Glycoprotein		15	34,1	6,0E-5
Angiogenesis		4	9,1	2,4E-4
Cell cycle		6	13,6	3,5E-4

HCC

	Genes	Count	%	P-Value
Ubl conjugation		18	31,0	4,8E-11
Apoptosis		12	20,7	1,9E-10
Phosphoprotein		30	51,7	1,1E-7
Disulfide bond		21	36,2	7,3E-7
Glycoprotein		18	31,0	3,6E-5
Signal		23	39,7	5,0E-5
Cytoplasm		18	31,0	1,7E-4
Nucleus		18	31,0	1,8E-4
Cyclin		4	6,9	2,5E-4
Developmental protein		8	13,8	4,0E-4

B Pathways

RH

	Genes	Count	%	P-Value
Pathways in cancer		48	57,1	4,7E-43
MicroRNAs in cancer		25	29,8	2,4E-24
Hepatitis B		23	27,4	1,0E-21
Prostate cancer		19	22,6	6,3E-20
PI3K-Akt signaling pathway		29	34,5	7,9E-20
Pancreatic cancer		17	20,2	2,7E-19
Colorectal cancer		16	19,0	1,1E-17
Bladder cancer		14	16,7	1,7E-17
Melanoma		16	19,0	6,2E-17
Proteoglycans in cancer		22	26,2	6,9E-17

HCC

	Genes	Count	%	P-Value
Pathways in cancer		32	55,2	3,7E-28
Hepatitis B		16	27,6	3,7E-15
MicroRNAs in cancer		16	27,6	5,7E-15
Bladder cancer		10	17,2	2,1E-12
PI3K-Akt signaling pathway		18	31,0	1,3E-11
Proteoglycans in cancer		15	25,9	1,6E-11
Prostate cancer		11	19,0	1,4E-10
Colorectal cancer		10	17,2	1,9E-10
Non-small cell lung cancer		9	15,5	1,8E-9
Apoptosis		9	15,5	4,2E-9

Supplementary Figure 6: Functional categories and Pathway analysis of differentially expressed genes. Top 10 enriched functions **A.** and pathways **B.** in RH cells and rat HCC. *P* values were determined using the DAVID's EASE Score and were judged significant at $P < 0.05$.

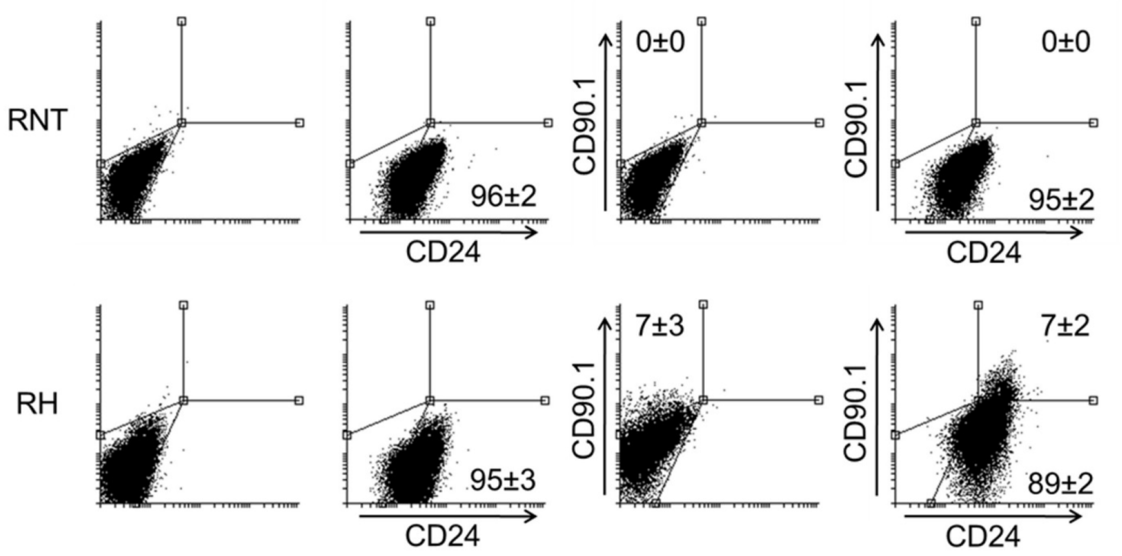

Supplementary Figure 7: Flow cytometry analysis of freshly isolated CD24+ RNT and RH cells. Isolated cells were highly positive for CD24 (≥95%). A subpopulation of CD90.1+ cells (7±2%) is present within the CD24+ RH cells, while no CD90.1+ cells were found in CD24+ RNT cells. Numbers represent percentage of positivity ± SD of 5 flow cytometry analysis from several isolations.

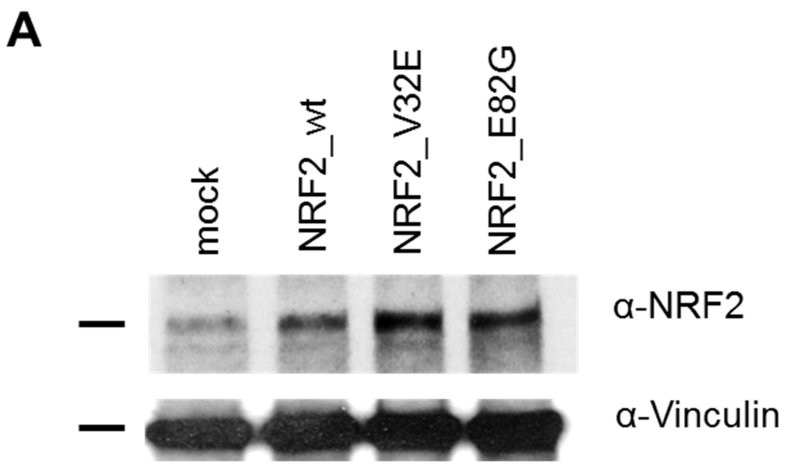

Supplementary Figure 8: Western blot analysis of RNT cells transduced with mock, NRF2 wild type or mutated NRF2. The blot was probed with NRF2 antibodies (upper panel). Vinculin staining (lower panel) was used as loading control.

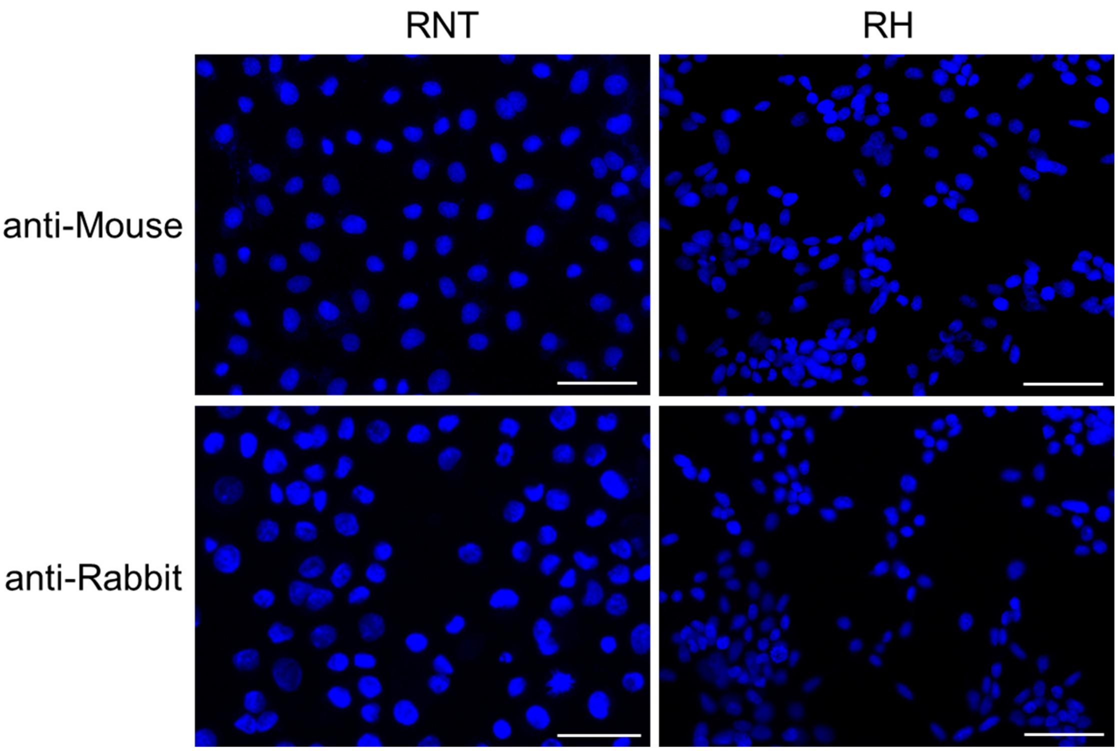

Supplementary Figure 9: Negative controls for immunofluorescence on RNT and RH cells. RNT and RH cells were stained only with fluorochrome-conjugated secondary anti-mouse or anti-rabbit antibodies (Alexa Fluor® 488 or Alexa Fluor® 546). Nuclei were stained with DAPI. Bars = 50 µm.

Supplementary Table 1: Analysis of gene expression in RH cells

Symbol	Fold Change	Symbol	Fold Change	Symbol	Fold Change
Adam17	0,68	Fas	0,02	Ptgs2	0,00
Akt1	1,42	Fhit	3,26	Ptk2	1,40
Angpt2	1,80	Flt1	0,00	Pycard	0,41
Bax	0,79	Fzd7	3,86	Rac1	0,60
Bcl2	0,92	Gadd45b	1,36	Rassf1	0,72
Bcl2l1	2,05	Gstp1	1,71	Rb1	1,81
Bid	4,93	Hgf	41,94	Reln	0,58
Birc2	1,41	Hhip	2,26	Rhoa	0,97
Birc5	3,07	Hras	1,57	Runx3	411,03
Casp8	0,26	Igf2	0,45	Sfrp2	0,00
Ccl5	1,02	Igfbp1	2,26	Smad4	0,93
Ccnd1	0,57	Igfbp3	291,95	Smad7	0,59
Ccnd2	0,26	Irs1	0,27	Socs1	2,59
Cdh1	2,24	Itgb1	1,25	Socs3	5,36
Cdh13	0,67	Kdr	3,11	Stat3	2,57
Cdkn1a	0,22	Lef1	0,03	Tcf4	0,05
Cdkn1b	0,72	Mcl1	1,17	Tert	0,14
Cdkn2a	34819,92	Met	1,41	Tgfa	10,60
Cflar	0,54	Msh2	2,04	Tgfb1	0,02
Ctnnb1	1,06	Msh3	0,59	Tgfbr2	1,35
Cxcr4	0,16	Mtdh	1,91	Tlr4	0,55
Dab2ip	0,03	Myc	2,71	Tnfrsf10b	15,03
Dlc1	0,53	Nfkb1	0,78	Tnfsf10	2,26
E2f1	0,63	Nras	1,25	Tp53	0,04
Egf	2,64	Opcml	167,63	Vegfa	6,10
Egfr	0,02	Pdgfra	0,02	Wt1	1,14
Ep300	1,84	Pin1	1,20	Xiap	1,68
Fadd	0,93	Pten	0,37	Yap1	1,03

Gene expression is reported as fold change relative to RNT cells.

Supplementary Table 2: Antibodies used for Immunofluorescence

Primary antibody			
**Name**	**Conjugation**	**Brand**	**Incubation conditions**
anti-alpha-SMA	Purified	Sigma Aldrich	60 min at RT
anti-rat Tie2	Purified	Santa Cruz Biotec	60 min at RT
anti-Desmin	Purified	Abcam	60 min at RT
anti-Vimentin	Purified	Sigma Aldrich	60 min at RT
anti-KTR18	Purified	Santa Cruz Biotec	60 min at RT
anti-KTR19	Purified	Novus Biologicals	60 min at RT
anti-Albumin	Purified	Santa Cruz Biotec	60 min at RT
anti-Transferrin	Purified	Abcam	60 min at RT
anti-HNF4α	Purified	Abcam	60 min at RT
anti-TTR	Purified	Thermo Fisher Scientific	60 min at RT
**Secondary antibody**			
**Name**	**Conjugation**	**Brand**	**Incubation conditions**
Goat anti-rabbit	Alexa Fluor488 or 546	Life Technologies	45 min at RT
Goat anti-mouse	Alexa Fluor488 or 546	Life Technologies	45 min at RT

Supplementary Table 3: Antibody used for flow cytometry

Primary antibody			
**Name**	**Conjugation**	**Brand**	**Incubation conditions**
anti-rat CD24	PE	MiltenyiBiotec	30 min on ice
anti-rat CD90.1	APC	MiltenyiBiotec	30 min on ice
anti-rat EpCAM	PE	Santa Cruz Biotech	30 min on ice
anti-rat Albumin	FITC	Abcam	30 min on ice
anti-rat KRT18	PE	Santa Cruz Biotech	30 min on ice
anti-rat KRT19	Alexa Fluor 488	Novus Biologicals	30 min on ice
anti-rat CD68	FITC	MiltenyiBiotec	30 min on ice
anti-alpha-SMA	Purified	Sigma Aldrich	30 min on ice
anti-Desmin	Purified	Abcam	30 min on ice
anti-Vimentin	Purified	Sigma Aldrich	30 min on ice
**Secondary antibody**			
**Name**	**Conjugation**	**Brand**	**Incubation conditions**
Goat anti-rabbit	Alexa Fluor488 or 546	Life Technologies	30 min on ice
Goat anti-mouse	Alexa Fluor488 or 546	Life Technologies	30 min on ice
